# Supplementary material for: Synthesis of Novel Shape Memory Thermoplastic Polyurethanes (SMTPUs) from Bio-Based Materials for Application in 3D/4D Printing Filaments
Source: Materials (Basel). 2023 Jan 26;16(3):1072. doi: 10.3390/ma16031072 (PMC9921888; doi:10.3390/ma16031072)
Supplement: Supplementary file 1 [file materials-16-01072-s001.zip › materials-2145758-supplementary.pdf]

Synthesis of Novel Shape Memory Thermoplastic Polyurethanes (SMTPUs) from Bio-based Materials for Application in 4D Printing Filaments

Yang-Sook Jung[1,2], Sunhee Lee[3], Jaehyeung Park [2*] and Eun-Joo Shin[1*]

[1] Department of Organic Materials and Polymer Engineering, Dong-A University, Busan 49315, Republic of Korea

[2] Department of Bio-fibers and Materials Science, Kyungpook National University, Daegu 41566, Republic of Korea

[3] Department of Fashion Design, Dong-A University, Busan 49315, Republic of Korea

* Correspondence: parkj@knu.ac.kr (J.P.); sejoo6313@dau.ac.kr (E.J.S.); Tel.: +82-539505738 (J.P.); +82-512007343 (E.J.S.)

Supporting Information

Table S1. Photographic images of the loading set-up and the resulting products during the preliminary test before 3D printing.

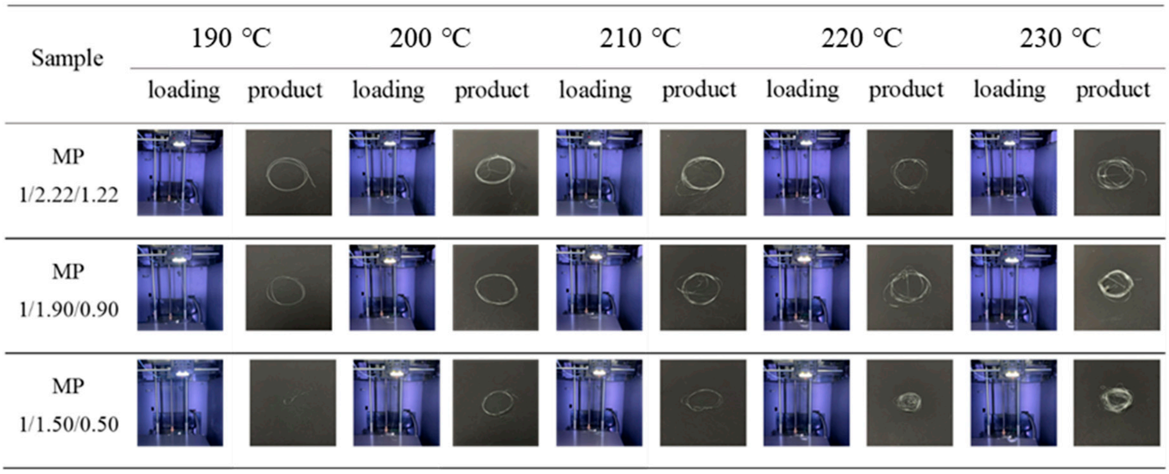

Sample	190 °C		200 °C		210 °C		220 °C		230 °C	
	loading	product	loading	product	loading	product	loading	product	loading	product
MP 1/2.22/1.22										
MP 1/1.90/0.90										
MP 1/1.50/0.50										

Table S2. Images of the auxetic re-entrant shape memory thermoplastic polyurethane samples.

Sample	MP 1/2.22/1.22	MP 1/1.90/0.90	MP 1/1.50/0.50
Image	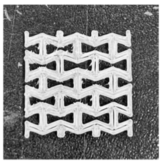	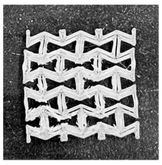	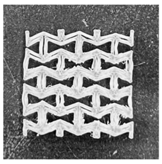
Nozzle Temperature (°C)	210	230	230

Weight (g)	0.70	0.74	0.67
